# INTIMATE RELATIONSHIPS, SOCIAL ISOLATION, AND COGNITION

**DOI:** 10.1093/geroni/igad104.0897

**Published:** 2023-12-21

**Authors:** Michal Engelman, Sara Moorman

## Abstract

This symposium considers the cognitive impacts of a spectrum of social relationships, including both intimate partnerships and, in contrast, social isolation. Drawing on rich data from the Wisconsin Longitudinal Study (WLS), the Health and Retirement Study (HRS), the National Social Life, Health, and Aging Project (NSHAP), and the Mexican Health and Aging Study (MHAS), the papers showcase empirical linkages between older adults’ cognitive health with a set of exposures including marital status and relationship characteristics, social activities, and a lack of social connections. We begin with papers that examine the impact of particular marital relationship attributes and social behaviors on cognition and health. One paper employs the WLS to explore the impact of concordance and discordance in marital couples’ reports of similarity with and closeness to their spouse on cognition, while the second draws on NSHAP to consider the cognitive consequences of various domains of couples’ sexuality. The third paper uses the HRS to examine couples’ co-trajectories of physical, mental, and cognitive health, focusing particularly on how a spouse’s cognitive decline influences the other partner’s subsequent health trajectory. The fourth paper shifts our attention to variation by marital status in the cognitive impacts of leisure activities via data from MHAS, while the fifth focuses specifically socially-isolated older adults in the HRS and highlights variation in the relationship between social isolation and dementia by race and ethnicity. The symposium will conclude with a discussion of future directions for research on the cognitive consequences of relationships, social connections, and social isolation.

